# Supplementary material for: Deciphering the Complex Intertwining Between Cytopenia and Transfusion Needs After CAR-T-Cell Therapy for B-Cell Malignancies
Source: Life (Basel). 2025 Sep 9;15(9):1419. doi: 10.3390/life15091419 (PMC12471091; doi:10.3390/life15091419)
Supplement: Supplementary file 1 [file life-15-01419-s001.zip › life-3805636-supplementary.pdf]

Supplementary Table S1

	Early transfusion needs (< 1 month)		Late transfusion needs (≥ 1 month)	
	RBC	***Platelets***	***RBC***	***Platelets***
Age > 60 years	*0.733*	*0.250*	*0.603*	0.968
ECOG PS ≥ 2	*0.493*	**0.008**	*0.869*	0.389
Female sex	**< 0.001**	0.537	*0.621*	0.648
Disease histology	*0.674*	*0.142*	*0.333*	0.294
Prior lines > 3	***0.033***	***0.005***	*0.491*	0.791
ASCT	*0.113*	*0.205*	**0.001**	**0.007**
Reduced BM cellularity	***< 0.001***	***0.013***	0.05	0.139
BM infiltration	*0.319*	0.482	0.482	0.909
Progressive disease	***0.007***	0.327	0.124	0.260
Pre CAR-T cell RBC units*	***<0.001***	**0.001**	**<0.001**	**<0.001**
Pre CAR-T cell platelet units*	*0,003*	**<0.001**	**<0.001**	**<0.001**
Any transfusion pre-CAR-T	***< 0.001***	***< 0.001***	***< 0.001***	***< 0.001***
CAR-HEMATOTOX High risk	***< 0.001***	**0.002**	0.176	0.342
CAR-T Product	0.347	0.113	0.531	0.932
CAR T-cells expansion peak value	0.290	0.543	0.994	0.856
CAR-T AUC (0-14d)<br>CAR-T AUC (0-30d)	0,138<br>0,179	0.552<br>0.968	0.922<br>0.850	0.628<br>0.595
CRS incidence	0.076	0.141	0.141	0.174
CRS grade >2	**0.002**	0.138	**0.034**	0.101
Serum IL-6 peak	**0.021**	0.749	0.164	0.077
Serum IL-2R peak	**0.038**	0.467	0.261	0.575
Tocilizumab	0.264	0.926	0.203	0.347
Tocilizumab doses	0.083	0.837	0.257	0.684
ICANS	0.178	**0.006**	0.845	0.410
ICANS >2	0.168	***< 0.001***	0.379	0.252
Early ICAHT	***< 0.001***	**0.003**	**0.024**	**0.007**
Early ICAHT>2	***< 0.001***	**0.001**	0.148	**0.011**
Late ICAHT	0.050	**<0.001**	**<0.001**	**<0.001**
Late ICAHT >2	**0.003**	**<0.001**	**<0.001**	**<0.001**

Table S1: Univariate analysis

*Transfused in the three months before CAR T-cell therapy

Other factors tested in the univariate analysis but not significant were: number of prior lines, CAR-T expansion days 7-14-30, Neutropenia ≥ G3, neutropenia duration (days).

List of abbreviations: **ASCT:** autologous hematopoietic stem cells transplantation; **AUC:** area under the curve**; BM:** bone marrow**; CAR-T:** Chimeric Antigens Receptor Cells-T; **ECOG PS:** Eastern Cooperative Oncology Group Performance Status; **ICANS:** Immune effector cell-associated neurotoxicity syndrome; **ICAHT:** immune effector cell–associated hematotoxicity; **IL-6:** interleukin 6**; IL-2R:** interleukin-2 receptor; **RBC:** red blood cells.

Supplementary Figures

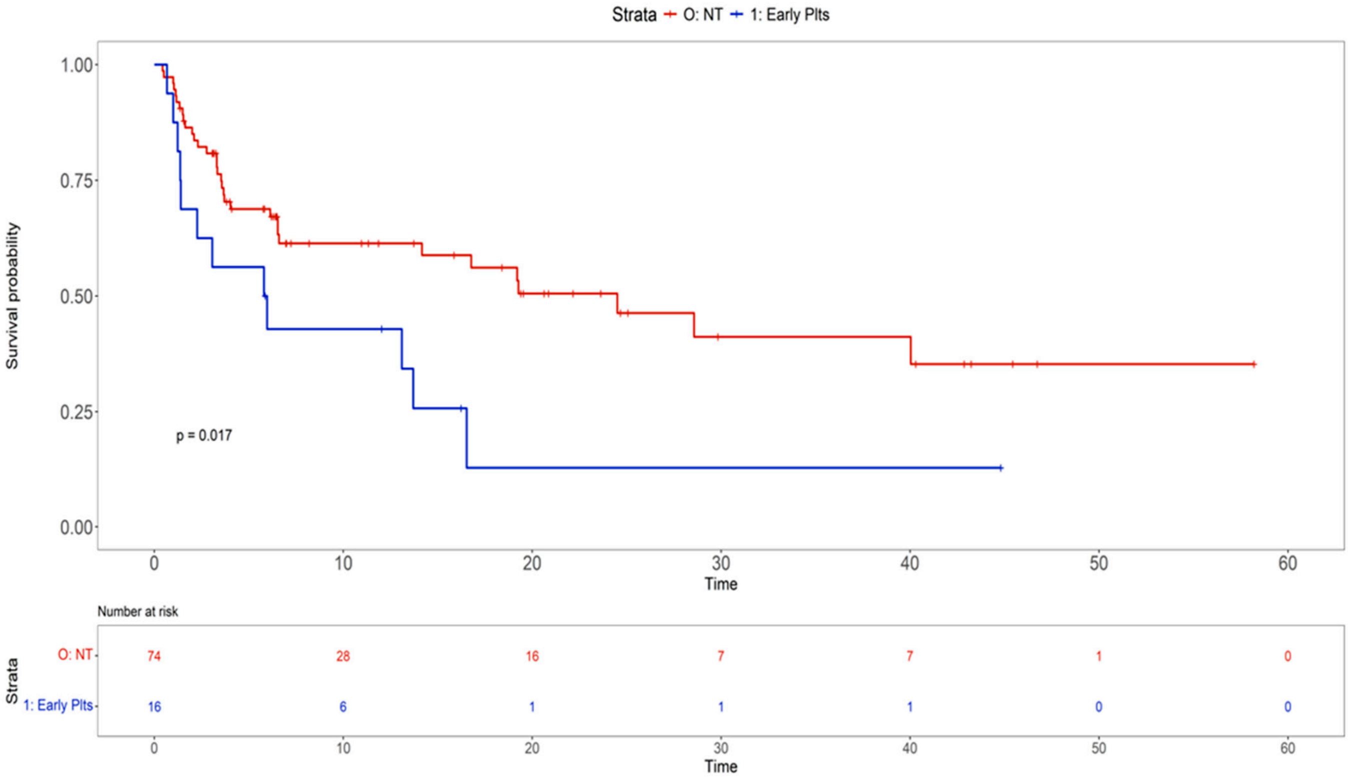

Figure S1: Comparison of progression-free survival in patients receiving at least one Plt transfusion in the first month post-CART-cell and those who did not

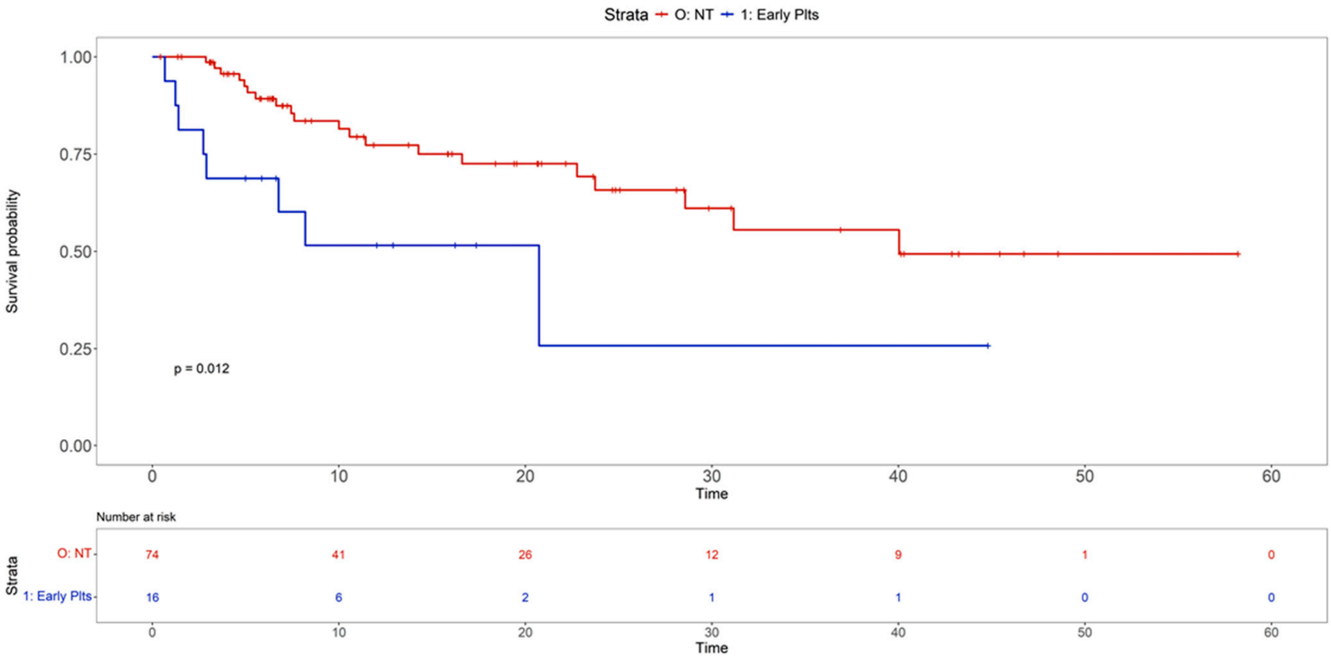

Figure S2: Comparison of overall survival in patients receiving at least one Plt transfusion in the first month post-CART-cell and those who did not

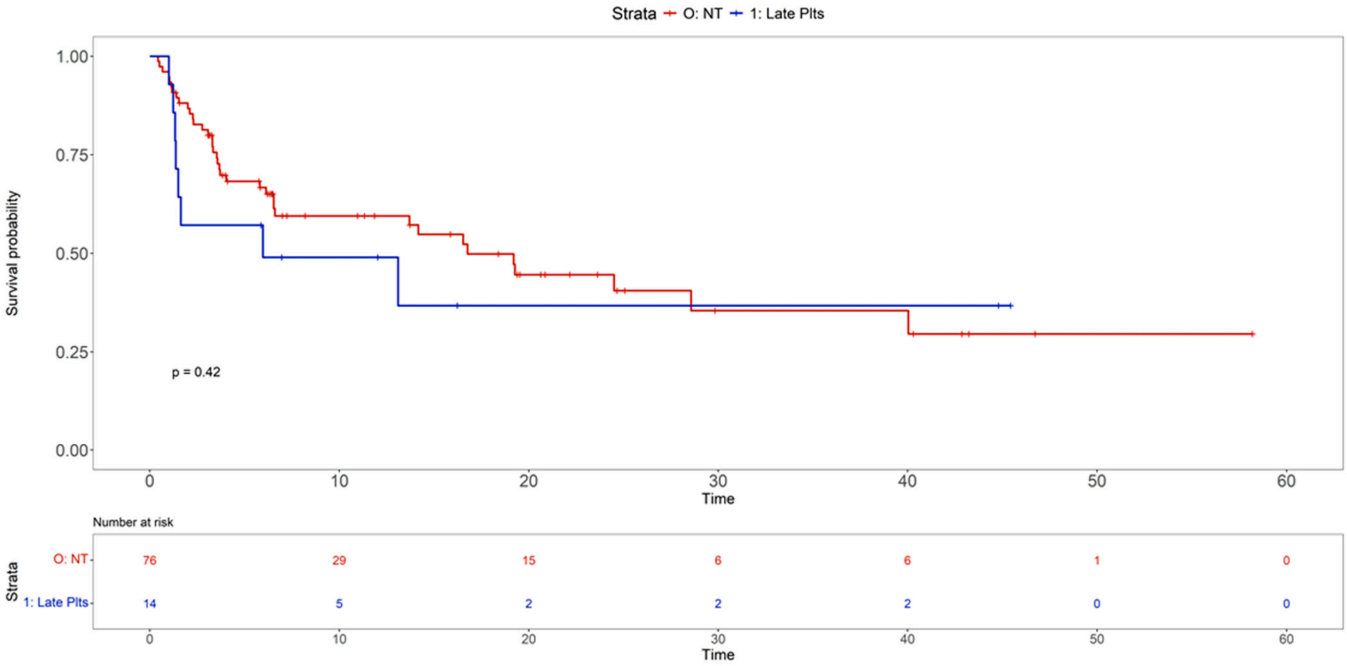

Figure S3: Comparison of progression-free survival in patients receiving at least one Plt transfusion in months 2-3 post-CART-cell and those who did not

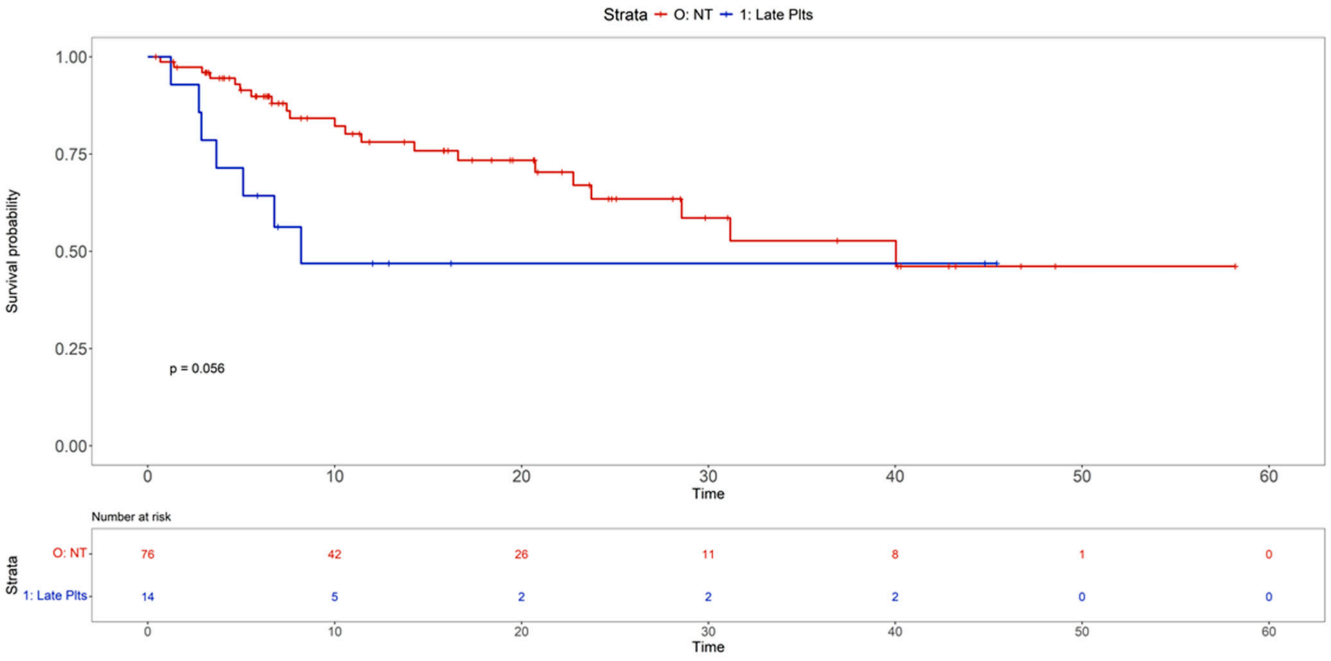

Figure S4: Comparison of overall survival in patients receiving at least one Plt transfusion in months 2-3 post-CART-cell and those who did not

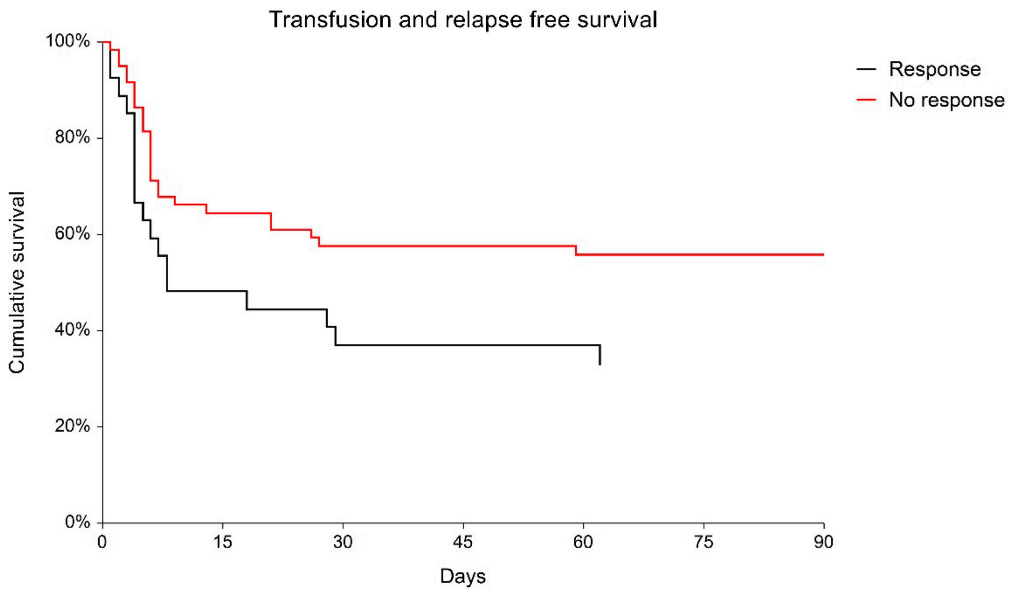

Figure S5: Transfusion-free survival (TRFS) after CAR T-cells infusion according to overall response

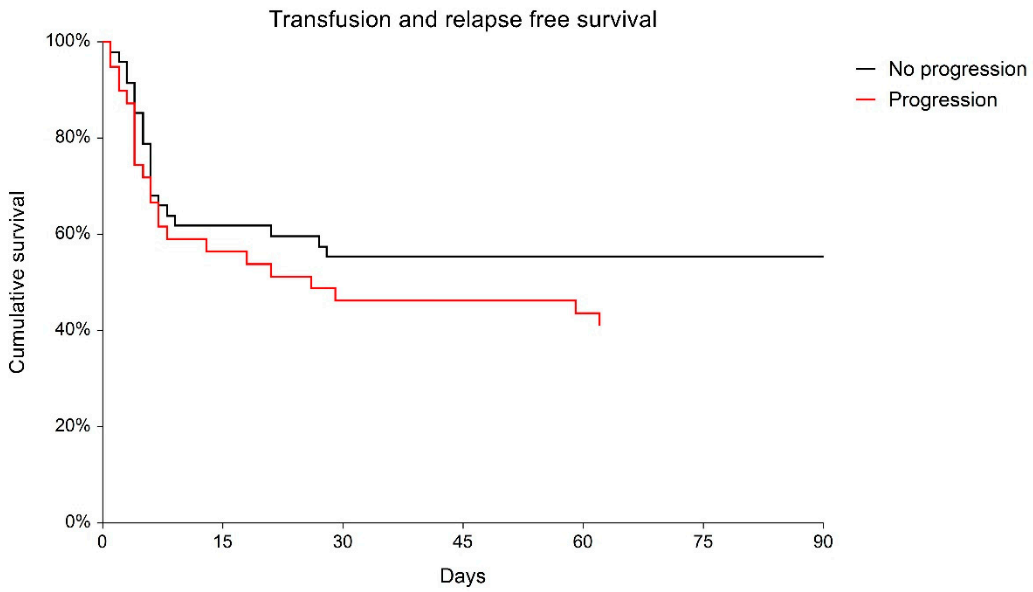

Figure S6: Transfusion-free survival (TRFS) after CAR T-cells infusion according to disease progression

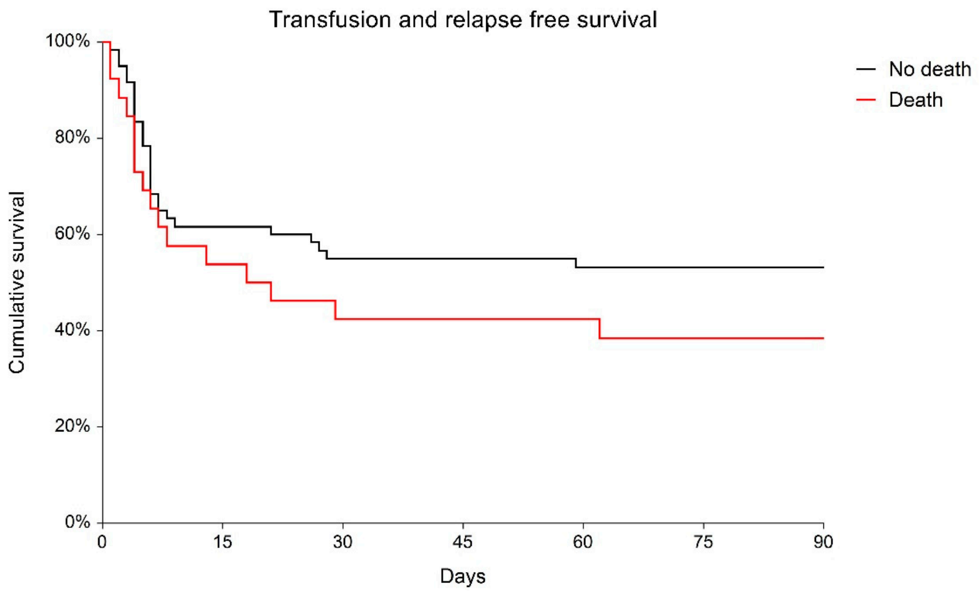

Figure S7: Transfusion-free survival (TRFS) after CAR T-cells infusion according to death

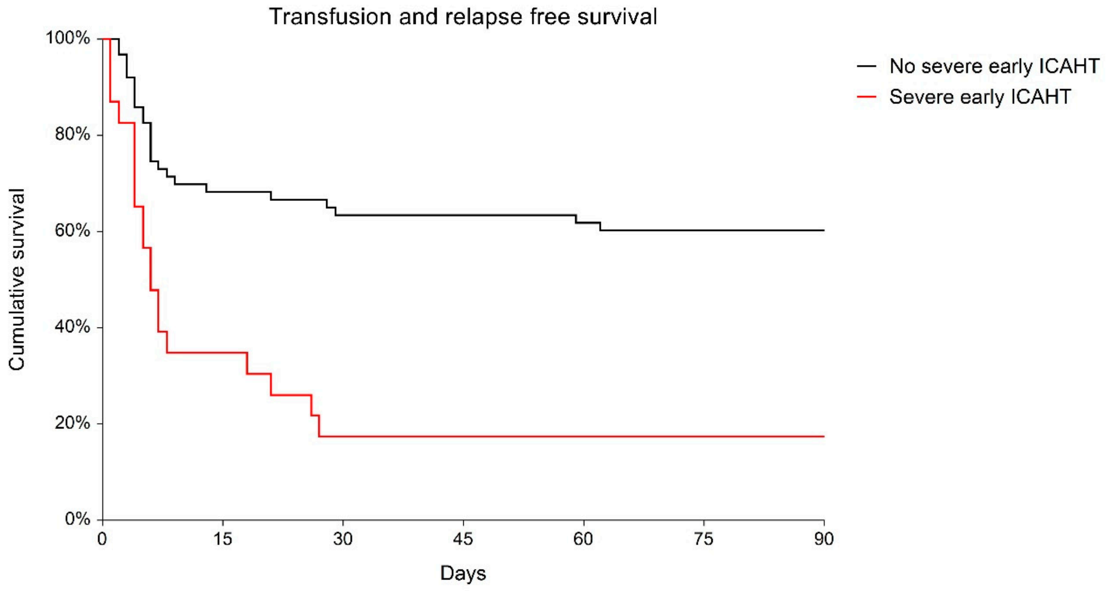

Figure S8: Transfusion-free survival (**TRFS**) after CAR T-cells infusion according to the development of severe early ICAHT

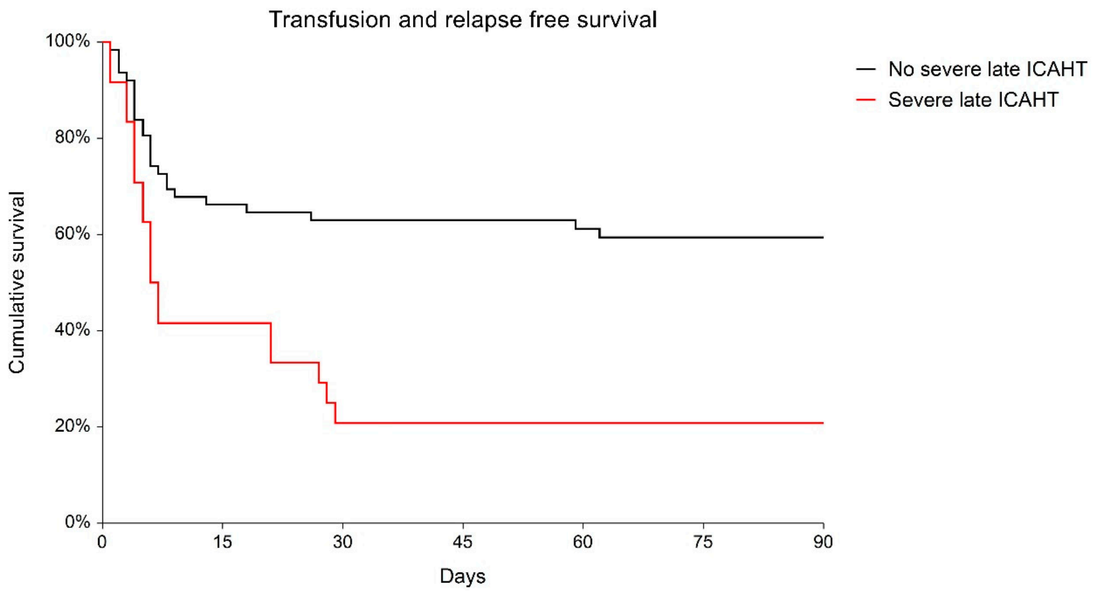

Figure S9: Transfusion-free survival (**TRFS**) after CAR T-cells infusion according to the development of severe late ICAHT
